# Bi-national outbreak of *Salmonella* Newport infections linked to onions: the United States experience

**DOI:** 10.1017/S0950268822001571

**Published:** 2022-11-16

**Authors:** Z. D. McCormic, K. Patel, J. Higa, J. Bancroft, D. Donovan, L. Edwards, J. Cheng, B. Adcock, C. Bond, E. Pereira, M. Doyle, M. E. Wise, L. Gieraltowski, the Salmonella Newport Investigation Team

**Affiliations:** 1 Centers for Disease Control and Prevention, Atlanta, USA; 2 Oak Ridge Institute for Science and Education, Oak Ridge, USA; 3 California Department of Public Health, Los Angeles, USA; 4 Oregon Health Authority, Salem, USA; 5 Michigan Department of Health and Human Services, Lansing, USA; 6 Public Health Agency of Canada, Ottawa, Canada; 7 US Food and Drug Administration, College Park, USA; 8 San Francisco Department of Public Health, San Francisco, USA

**Keywords:** Food-borne infections, outbreaks, salmonellosis

## Abstract

From 2016–2019, dry bulb onions were the suspected cause of three multistate outbreaks in the United States. We investigated a large multistate outbreak of *Salmonella* Newport infections that caused illnesses in both the United States and Canada in 2020. Epidemiologic, laboratory and traceback investigations were conducted to determine the source of the infections, and data were shared among U.S. and Canadian public health officials. We identified 1127 U.S. illnesses from 48 states with illness onset dates ranging from 19 June to 11 September 2020. Sixty-six per cent of ill people reported consuming red onions in the week before illness onset. Thirty-five illness sub-clusters were identified during the investigation and seventy-four per cent of sub-clusters served red onions to customers during the exposure period. Traceback for the source of onions in illness sub-clusters identified a common onion grower in Bakersfield, CA as the source of red onions, and onions were recalled at this time. Although other strains of *Salmonella* Newport were identified in environmental samples collected at the Bakersfield, CA grower, extensive environmental and product testing did not yield the outbreak strain. This was the third largest U.S. foodborne *Salmonella* outbreak in the last 30 years. It is the first U.S. multistate outbreak with a confirmed link to dry bulb onions, and it was nearly 10-fold larger than prior outbreaks with a suspected link to onions. This outbreak is notable for its size and scope, as well as the international data sharing that led to implication of red onions as the primary cause of the outbreak. Although an environmental assessment at the grower identified several factors that likely contributed to the outbreak, no main reason was identified. The expedient identification of the outbreak vehicle and response of multiple public health agencies allowed for recall and removal of product from the marketplace, and rapid messaging to both the public and industry on actions to protect consumers; these features contributed to a decrease in cases and expeditious conclusion of the outbreak.

## Background

*Salmonella* infections are a leading cause of bacterial gastroenteritis in the United States, causing an estimated 1 million illnesses and 400 deaths annually [1]. *Salmonella* is the second leading cause of foodborne outbreaks and produce-associated salmonellosis outbreaks are common. Recently, the percentage of outbreaks attributed to raw produce has increased [2]. However, raw produce outbreaks associated with bulb-style root vegetables, such as onions and garlic, are rare. Approximately 1% of produce-associated outbreaks have been attributed to bulb vegetables [2]. Since 2016, three multistate outbreaks of *Salmonella* have had a suspected link to dry bulb onions; all three outbreaks were caused by *Salmonella enterica* serovar Javiana [3]. These outbreaks varied in size from 29 to 149 ill people, and were all identified between August and September of their respective years.

Raw produce-associated outbreaks can be challenging to investigate. Raw produce items are subject to multiple potential contamination points, including in the field, during harvest, packing or processing, transportation, storage and during final preparation [2]. Raw produce items can be served in a variety of dishes, such as in salads and on sandwiches which often contain various other raw produce ingredients that may be difficult for ill people to remember. Traceback of raw produce items to their field of origin, particularly for those that can be purchased in bulk and without labelling, can be difficult [4]. Finally, the short shelf-life of many raw produce items can limit opportunities for testing leftover foods to identify the outbreak strain.

*Salmonella enterica* serovar Newport is one of the five most common *Salmonella enterica* serovars reported in the United States [5]. Past outbreaks of *Salmonella* Newport have been associated with a variety of foods, including beef, cheese [6], cucumbers [7] and tomatoes [8]. In this report, we describe the U.S. investigational activities related to this bi-national *Salmonella* Newport outbreak.

## Methods

### Case-finding and case definition

State and local public health laboratories received and sequenced *Salmonella* isolates from ill people using whole genome sequencing (WGS) by standardised methods [8]. Public health laboratories submitted WGS information to PulseNet, the national molecular subtyping network for foodborne disease surveillance [9], which then conducted WGS analyses using BioNumerics version 7.6 [10]. For comparison of strains sequenced by WGS, PulseNet primarily uses an allele-based, core genome multi-locus sequence typing (cgMLST) scheme incorporated into the Salmonella national database. This allele scheme contains 3002 loci developed from the publicly available and actively curated databases [11, 12]. For *Salmonella* cluster detection, PulseNet uses a threshold of 3 or more clinical cases relating within 0–10 cgMLST allele differences. On 9 July 2020, PulseNet identified a cluster of 10 isolates from ill people in 4 states that differed by 0 alleles from one another by cgMLST analysis. By the end of the investigation, any isolate within 0–6 allele differences by cgMLST was considered to be of the outbreak strain. For this investigation, we defined a case as laboratory-confirmed infection with the outbreak strain of *Salmonella* Newport with illness onset between 15 June 2020 and 11 September 2020.

### Epidemiologic investigation

Ill people were initially interviewed with routine state enteric illness questionnaires or with the National Hypothesis Generating Questionnaire [13], which includes questions on over 200 food and other exposures in the week before illness onset. Based on results of these initial interviews, ill people were asked more detailed questions about suspected food items, such as purchase location, brand and purchase date, and data were collected using the System for Enteric Disease Response, Investigation and Coordination (SEDRIC). Through interviews, we identified illness sub-clusters in which two or more people not living in the same household reported eating at the same restaurant location, attending a common event or shopping at the same location of a grocery store in the week before becoming ill. For facilities associated with sub-clusters, invoices were requested and reviewed for the presence of suspected food items and for documenting details such as type and variety. Analysis of invoices focused on identifying common foods received at sub-cluster locations during the period of interest, defined by the purchase and consumption dates of ill people associated with the sub-cluster.

Concurrently, we learned that Canada was investigating clinical isolates from ill people in two Canadian provinces that were closely related by WGS to clinical isolates in the U.S. cluster on 20 July 2020. Because rapidly growing outbreaks with an unidentified source were occurring in both countries, the United States and Canada began routinely sharing epidemiologic and other investigational data, including exposure information for ill people and information on illness sub-clusters.

### Traceback investigations

The U.S. Food and Drug Administration (FDA) and local and state regulatory agencies, including the California Department of Public Health (CDPH) and California Department of Food and Agriculture (CDFA), conducted traceback activities to identify common suppliers of suspect food items. Traceback activities are defined as the ‘process of reviewing product supply chain records to identify the origin of food served or sold at a specific point of service (POS)’ [4]. Information related to ill people's food exposures, including purchase dates and locations were collected during sub-cluster investigations and used to determine the ultimate source(s) of foods supplied to the point of sale. Sub-clusters prioritised for traceback included those with more illnesses and with known meal or exposure dates. Records including receipts, invoices, bills of lading and other relevant product traceability documents were obtained from restaurants associated with sub-clusters. A traceback leg was defined as the ‘supply chain for a specific illness sub-cluster or single case exposure, initiated at the POS.’ [4]

### Field investigations

FDA, CDPH and CDFA investigators conducted on-farm investigations and sample collections at locations identified in traceback, including farms, packing facility, public lands and canals. Investigators conducted record reviews, interviews, and made observations on environmental factors, adjacent land use, and growing and harvesting practices (*e.g*., planting, irrigation, soil amendments, water, animal activity). Specimens collected included water, sediment, scat, environmental and product samples during field investigations at the identified producer and product samples from distribution centres. Additional food samples were collected by state investigators. Food and environmental samples were analysed according to the Bacteriological Analytical Manual *Salmonella* culture method [14]. Presumptive colonies were confirmed as *Salmonella* using these validated techniques [14].

## Results

### Case finding

**Fig. 1.**
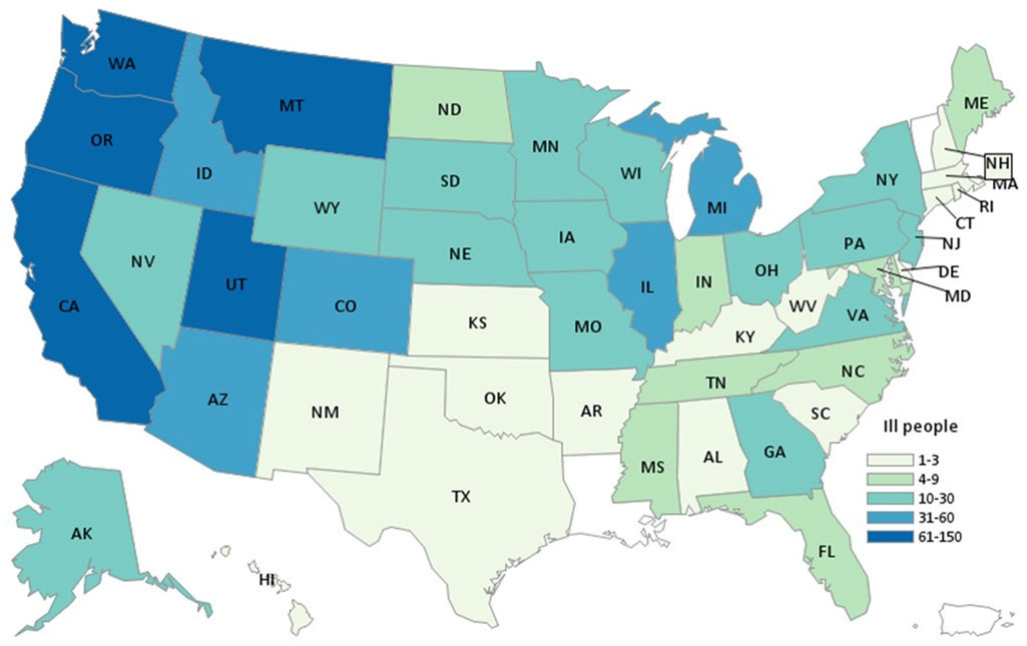
Map of residence of reported ill persons. Summary: People infected with the outbreak strain of *Salmonella* Newport (*n* = 1127) by state of residence, United States, 2020.

**Fig. 2.**
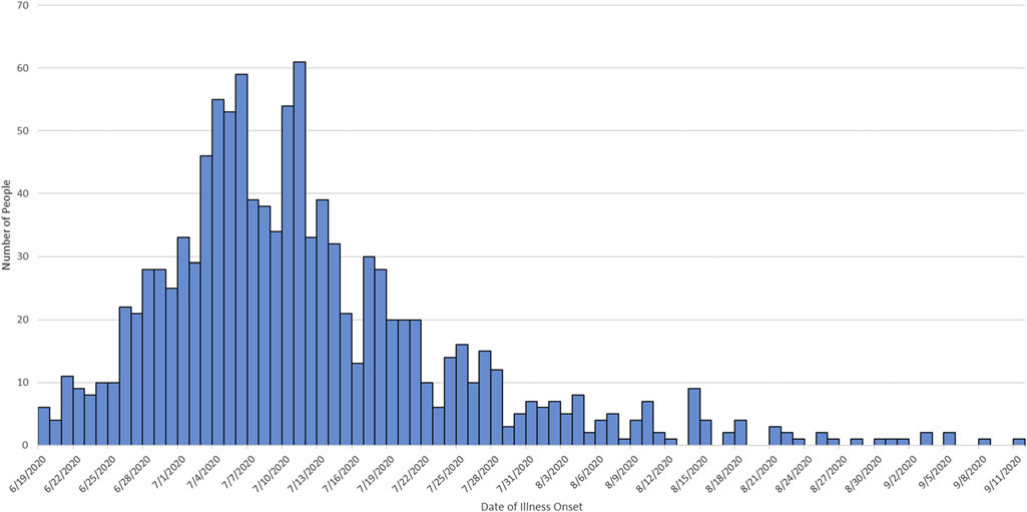
Epidemic curve of reported illnesses by onset date. Summary: People infected with the outbreak strain of *Salmonella* Newport (*n* = 1127) by date of illness onset, United States, 2020. Some illness onset dates have been estimated from other reported information.

During the investigation we identified 1127 ill people in the United States with *Salmonella* Newport infections with the outbreak strain. Ill people were identified in 48 states (Fig. 1); Washington (*n* = 150) and California (*n* = 128) reported the highest numbers of ill people. Illness onset dates ranged from 19 June 2020 to 11 September 2020 (Fig. 2). The median age of ill people was 41 years (range of 5 days to 102 years) and 58% were female. Twenty-four per cent (167/705) were hospitalised, and no deaths were reported.

All sequences from clinical isolates in the outbreak were closely related to each other by cgMLST (within 0 to 6 allele differences). The 2020 outbreak strain was also closely related (within 0 to 5 allele differences) to a historical illness cluster of 36 ill people investigated in the fall of 2015, for which no suspect vehicle had been identified. The National Center for Biotechnology Information's Pathogen Detection Pipeline [15] showed that the non-clinical isolate sequence most closely related to the outbreak strain was an almond isolate collected in 2017 from California which differed by up to 14 single nucleotide polymorphisms.

### Epidemiologic investigation

We analysed detailed epidemiologic data provided by 28 states on 380 ill people, including information from 35 illness sub-clusters in 14 states. Initial information gathered from ill people revealed several commonly reported produce items. Early analysis of data from 305 ill people showed that 90% reported any onion consumption in the 7 days before illness onset. Additionally, 78% reported tomatoes, 75% any leafy greens, 48% cilantro, 42% any bell peppers and 16% any chili peppers. Tomato, cilantro and leafy green exposure were reported significantly more frequently than expected among ill people in the outbreak compared to the frequency reported among healthy adults in the U.S. FoodNet Population Survey (*P* < 0.001 for all 3 comparisons) [16]; no individual type of leafy green was reported significantly higher than expected. Most illness sub-clusters in the United States were at restaurants serving Mexican-style cuisine, and included ill people with exposure to several common produce items. Tomatoes, onions, cilantro, peppers and leafy greens were hypotheses considered during this phase of the investigation, based on the food histories of ill people and the illness sub-cluster data.

We also evaluated onion consumption by onion type. Among 380 ill people with detailed exposure data available, 208 had information on the types of onions they consumed: 66% reported red onions, 63% white onions and 53% yellow onions in the week before illness onset. Since there is no specific question for red onion exposure included in the U.S. FoodNet Population Survey [16], we utilised red onion exposure frequencies from outbreak investigations from 2013–2016 and the red onion consumption estimate from the Canada Foodbook Report [17]. These background exposure frequencies were 18.6% and 32.3%, respectively. Both indicated that the red onion exposure frequency of 66% was significantly higher than expected (*P* < 0.001 for both comparisons). Combined exposure to white or yellow onions among ill people in the outbreak, 78%, was significantly higher than the frequency in the U.S. FoodNet Population Survey [16], in which 71% of healthy people reported consuming yellow or white onions in the week before interview (*P* = 0.012). In total, 91% (344/380) of ill people reported eating red, white or yellow onions in the week before illness onset.

**Fig. 3.**
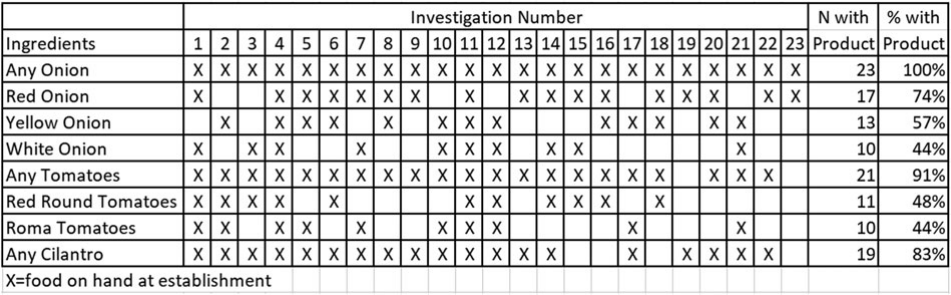
Ingredients by sub-cluster investigation. Select ingredients by sub-cluster investigation, total number of subclusters with product in-house, and per cent reporting product as part of *Salmonella* Newport investigation, United States, 2020. X indicates product was in-house at sub-cluster venue at time of ill person exposure.

We identified 35 illness sub-clusters in 14 states. Thirty sub-clusters were restaurant-associated or associated with a shared meal or event, and five were grocery store-associated. A review of invoices collected from 23 restaurant sub-clusters showed that onions of any type were present at all 23 (100%) restaurants (Fig. 3) during the period of interest. Seventeen (74%) restaurants received red onions, 13 (57%) received yellow onions and 10 (44%) received white onions. Twenty-one sub-clusters (91%) received tomatoes of several different varieties, including 11 (48%) that received red-round tomatoes and 10 (44%) that received Roma tomatoes. Additionally, 19 sub-clusters (83%) received cilantro.

In Canada, initially identified sub-clusters included ill people that reported consuming burgers or sandwiches at restaurants or in congregate living facilities. An analysis was conducted for 13 sub-clusters in Canada that shared a common supplier (5 restaurants and 8 congregate living facilities), which evaluated ingredients reported by ill people in the restaurant-associated sub-clusters. Thirteen (100%) sub-clusters received red onions, 11 (85%) received other bulb onions, 10 (77%) received green onions and 6 (46%) received cilantro. Thirteen (100%) sub-clusters received tomatoes; 10 (77%) received red round tomatoes, 9 (69%) received grape or cherry tomatoes and 6 (46%) received Roma tomatoes. In total, 48 sub-clusters were ultimately identified in the Canadian investigation.

### Onion traceback investigations

**Fig. 4.**
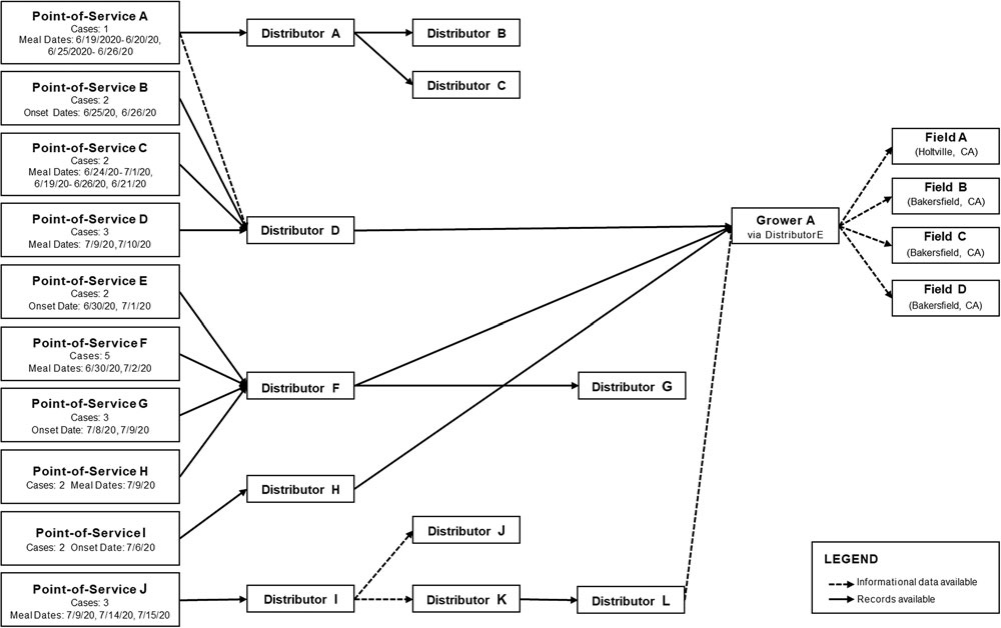
Traceback diagram of US cases. Traceback diagram of ill persons by Point of Service from investigation of *Salmonella* Newport, United States, 2020. Onions from 10 Points of Service traced to Grower A.

A regulatory traceback was initiated by FDA for ten POS in the United States (corresponding to 26 ill people) in four traceback legs representing four distribution chains (Fig. 4). Nine of the ten POS received red onions from one of three distribution centres of a common distributor, limiting some of the diversity of the traced legs. One POS received red onions from a separate distributor. All ten POS ultimately received red onions from a single source, an onion grower based in Bakersfield, CA, during the timeframe of interest. Based on available records, four field locations were identified as potentially growing the implicated red onions. In addition, CDPH conducted traceback of yellow onions from four additional POS (corresponding to 16 ill people), which ultimately showed that three of the four restaurants definitively received yellow onions from the same Bakersfield, CA-based grower that was identified in the FDA traceback.

Canadian officials initiated traceback of red onions for multiple clusters, including 9 of 13 illness sub-clusters mentioned previously. Red onions associated with the 9 sub-clusters were distributed by a common importer. A review of import documents identified a common vendor that was further linked to the same Bakersfield, CA-based grower. Subsequent traceback of other Canadian ill people determined that red onions linked to multiple illnesses were also from the same grower.

### Field investigations

Investigators were unable to directly observe the onion grower's two operations because growing, harvesting, packing and holding activities had ceased for the season. Records management, pest control and cleaning and sanitising procedures were discussed with the firm at the close of the investigations. Several potential contributing factors were assessed during the environmental assessment, including contaminated irrigation water, animal intrusion and water runoff from nearby livestock operations [18]. Though the investigations took place when harvesting and packing were not ongoing, standard procedures and logs from the harvest season were reviewed. Opportunities for *Salmonella* spread, including pests, animal intrusions and food contact surfaces that had not been cleaned, maintained or inspected as frequently as necessary were noted [18]. However, no main reason for the outbreak was identified [18].

As part of the field investigation, FDA, CDPH and CDFA collected 113 environmental samples from growing fields, the surrounding irrigation system, and from onion holding facilities along the distribution chain. Samples included 77 from onions or onion skins, as well as 13 water samples (7 ultra-filtration and 6 grab water), 11 sediment samples, 6 environmental samples from food contact surfaces, 5 soil or scat samples and 1 drag swab sample. *Salmonella* was identified in 10 samples: 5 from ultra-filtration water, 4 from sediment and 1 from scat. These 10 samples yielded 140 isolates which were analysed by WGS. A total of 17 different *Salmonella* strains were identified. A strain of *Salmonella* Newport was isolated from six of the ten samples, but they were not closely related genetically to the outbreak strain. *Salmonella* was not identified in the remaining 103 samples.

### Control measures

Advisories were issued on 21 July, 24 July and 31 July 2020 by multiple agencies, informing both the U.S. and Canadian public about the outbreak investigation [19–22]. On 1 August 2020, the grower voluntarily recalled red, yellow, white and sweet yellow onions. Recalled onions were sold in a variety of packages, under a variety of brands, and were distributed to all 50 states, the District of Columbia and Canada. Following the initial recall, dozens of additional recalls were issued for a variety of FDA- and USDA-regulated products due to the presence of recalled onions. Following the recall, the number of cases identified decreased, and the speed of cases identified slowed.

## Discussion

**Fig. 5.**
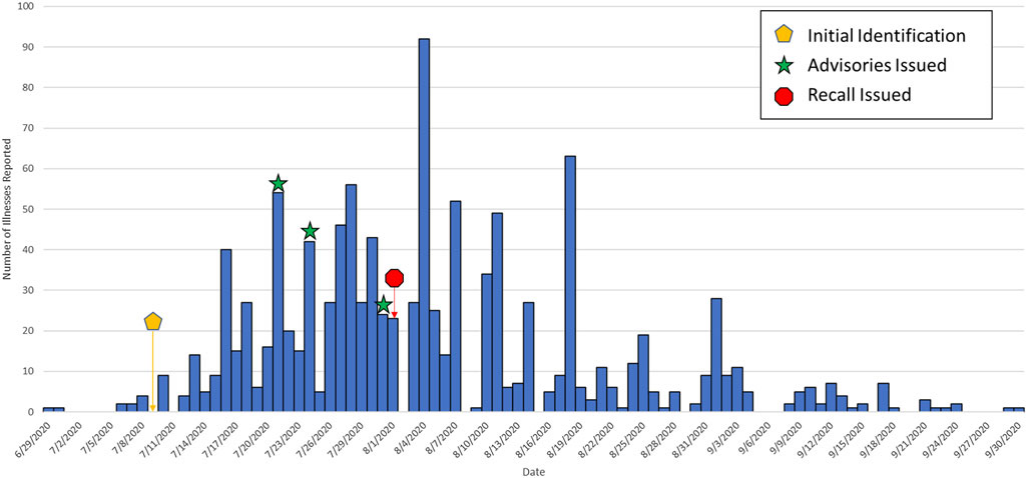
Timeline of key events and curve of reported illnesses by date of report. Timeline of key dates, including date investigation opened (orange pentagon), dates of outbreak-related advisories (green star) and the initial product recall (red octagon) with overlay of people infected with the outbreak strain of *Salmonella* Newport (*n* = 1127) by date of illness reported to CDC, United States, 2020.

This was the third largest U.S. multistate foodborne *Salmonella* outbreak since 1986 [3], and the largest in the era of whole genome sequencing. The outbreak grew rapidly, expanding from 10 illnesses reported at the time of detection to 508 illnesses at the time of the initial recall 23 days later. A timeline of key events and cases reported is available in Figure 5. Collaboration between local, state, federal and international public health agencies using epidemiological and traceback information resulted in the rapid identification of a vehicle and implementation of control measures to mitigate the outbreak, which led to a swift decrease in cases identified, and prevented additional illnesses from occurring. Prior to the recall and in the 2 weeks following the recall, 871 illnesses had been reported in 47 days since when the first illness was reported. We are including the two weeks after the recall into the cases before the recall, as these represent people who were ill from recalled product, but who had not yet been reported to public health. From this point until when the final case was reported, 256 illnesses were reported in 45 days. This represents a 69% decrease in the number of illnesses reported following the recall.

Raw produce-associated outbreaks are far less common than animal product-associated outbreaks [2]; among raw produce-associated outbreaks, outbreaks among root-style vegetables, a group which includes onions and garlic [23], are among the least common [24]. This outbreak was nearly ten-times larger than the largest prior outbreak with a suspected link to onions, a *Salmonella* Javiana outbreak in 2018 with 149 illnesses [3]. Given that outbreaks linked to onions are rare [24], an outbreak of this size and scope linked to dry bulb onions was not anticipated but is plausible. The on-site inspections at the grower did not identify any one main cause of onion contamination, but the following may have contributed to this historically large onion-associated outbreak. First, the sheer volume of onions produced by the implicated grower was high, and the number of different fields in which they were grown was relatively small. Therefore, contamination introduced into the growing or packing environment could impact a large number of onions. Second, irrigation water used on the onion fields came from a highly interconnected system of irrigation canals with many potential points of contamination. Irrigation water was not treated before use, hence if water were contaminated, it could have been applied directly to crops without an intervention to reduce microbial burden. Although the outbreak strain was not found in irrigation water, many other *Salmonella* strains were, highlighting the plausibility of this route of contamination. Third, all onions grown by the grower from all of the fields identified in the traceback investigation were harvested using the same equipment and processed in the same packing houses. The onion packing house was a dry operation, meaning water was not routinely used to clean and sanitise equipment [18]. Without routine wet equipment cleanings between lots of onions processed, onions were at risk of cross-contamination. Fourth, onions are generally not washed or cleaned during harvest and processing. According to a 2010 food safety guideline published by the National Onion Association, dry bulb onions should not be washed when removed from the ground, as this can lead to decay and mould, but that visible debris should be removed before processing [25]. Onions can be washed after the skin has been removed [25], but it is not known if this is routinely done in private homes or restaurants. Preparation, such as cutting, slicing and dicing, of contaminated onions may have increased the risk of cross contamination to cutting boards and knives. As a result, *Salmonella* bacteria could have contaminated other food items, even if the ill people did not report consuming any onions.

Sub-cluster investigations were key in identification of onions as the vehicle for this outbreak. Sub-cluster investigations allowed investigators to focus this large outbreak investigation on only those foods served at sub-cluster locations. A review of invoices from sub-clusters allowed investigators to rapidly find that red onions were supplied to three-quarters of the sub-cluster locations. Traceback of onions from these sub-cluster locations led to the identification of the common supplier of the red onions. The firm recalled multiple onion varieties because of the potential for cross-contamination between different varieties during growing, harvesting and packing. Interestingly, the sub-cluster locations that did not receive red onions did receive other dry bulb onion types. Although not definitive, this suggests that some illnesses in this outbreak could have been linked to exposure to other onion types.

Parallel, collaborative investigations were conducted in the United States and Canada. Throughout the investigation, we shared investigation-related materials, including food histories, leading hypotheses and traceback information. This collaboration led to a quicker identification of onions as the outbreak vehicle. Early hypothesis generation suggested a produce item, eaten raw, that was served as part of Mexican-style cuisine. Leafy greens, tomatoes, cilantro and onions were all considered in initial hypothesis generation. A thorough review of invoices collected from illness sub-clusters and joint analysis of exposure histories among ill people in Canada and the United States ruled out cilantro and tomatoes as the outbreak vehicle, and as a result, focused the investigation on red onions. Shared traceback information led to rapid identification of the supplier of onions to both countries, highlighting the advantages of binational collaboration. Following identification of the supplier, a large recall was initiated. After the recall, the number of cases identified decreased, and frequency of new cases being identified slowed considerably. Incorporating communications staff and discussions into routine bi-national calls facilitated alignment of public health messaging. This allowed all agencies to maintain consistent messaging via public outbreak notices when linking the outbreak to onions and advising people not to purchase or consume onions from the supplier. In the United States, the outbreak notice advice made national news headlines and the outbreak notice received over one million page views.

Timely data sharing was imperative during this investigation. In the United States, we utilise SEDRIC [26] to collate and analyse epidemiologic, laboratory and traceback information to streamline and coordinate outbreak investigations in real-time. Information from isolates, including demographic variables, are uploaded to SEDRIC through PulseNet. State and local partners can provide relevant clinical and epidemiological information related to these isolates, allowing data to be shared among local, state, federal and regulatory partners. We used SEDRIC to collect exposure information, including questionnaire data, along with traceback information, such as invoices, receipts or shopper card records. SEDRIC has proven an invaluable tool during outbreak investigations, as it provides a secure platform to accumulate investigation-related information and documents. Real-time data access is imperative to maximise limited resources during an outbreak investigation. Prior to SEDRIC, outbreaks of this size have taken hundreds of staff members to manage and multiple weeks to identify the vehicle of interest.

This outbreak investigation had several limitations. First, there was limited background data about red onion consumption in the United States. We utilised a case-case comparison from three prior produce-associated outbreaks in the United States, but case-case comparisons, especially using other outbreak-associated cases, can suffer from selection bias. Early in the investigation, we assessed overall onion exposure, rather than variety-specific, which led to a delay in establishing red onions as the cause of this outbreak. While consumption of any onion type was commonly reported, ultimately stratifying onion exposure by variety was needed to establish the association with red onions. Like all multistate foodborne outbreak investigations, ill people were often asked to recall food exposures that may have occurred weeks in the past. In this investigation, identification of specific onion type was a limiting factor, as ill people who consumed onions away from home had difficulty identifying the variety of onions they consumed, especially when included in multi-ingredient foods. Finally, federal, state and local agencies faced constraints due to the ongoing COVID-19 pandemic; resources were not always readily available for laboratory testing, WGS, timely case identification or interview of all ill people. As a result, the time from illness onset to interview for ill persons was longer than typical, which in turn may have increased recall bias.

Our traceback investigation was limited by the size of illness clusters identified, the lack of supply chain diversity (most sub-clusters were supplied by the same national distributor), and lack of adequate recordkeeping in the supply chain. Nine of ten sub-clusters identified for traceback had fewer than four ill people linked to a single POS. Smaller illness sub-clusters are more likely to represent chance findings in which the outbreak vehicle was not actually consumed at that venue. Three of the four legs of red onion traceback were through various distribution centres for a single company and did not represent a broad diversity of suppliers. Additionally, records were not available or were incomplete at some points along the distribution chains. Field-level information from the grower was inaccurate, making identification of suspected fields difficult. Finally, despite review and inspection at the grower, no main reason for the outbreak was identified.

This outbreak was the third largest multistate foodborne *Salmonella* outbreak since 1986, and the largest in the WGS era. Onions are a rare vehicle for *Salmonella* outbreaks in North America, and despite identification of several possible contributing factors, no main reason for this outbreak was identified. In 2021, CDC, FDA, state and local health departments investigated another large multistate outbreak linked to imported bulb onions [27], highlighting the urgent need to evaluate factors that may contribute to contamination of bulb onions and to develop interventions to reduce the likelihood of outbreaks and illnesses.

## Data Availability

If interested in accessing data or other materials related to this manuscript, readers may contact the corresponding author.
